# Predictive Models for Non-Alcoholic Fatty Liver Disease Diagnosis in Mexican Patients with Gallstone Disease: Sex-Specific Insights

**DOI:** 10.3390/diagnostics14141487

**Published:** 2024-07-11

**Authors:** Nemry Rodríguez-Hernández, María-Luisa Lazo-de-la-Vega-Monroy, Yeniley Ruiz-Noa, Monica-del-Carmen Preciado-Puga, Juana-Rosalba Garcia-Ramirez, Benjamin Jordan-Perez, Serafin Garnelo-Cabañas, Lorena-del-Rocío Ibarra-Reynoso

**Affiliations:** 1 Department of Medical Sciences, Health Sciences Division, University of Guanajuato, Leon Campus, Leon de los Aldama 37320, Mexico; n.rodriguezhernandez@ugto.mx (N.R.-H.); mlazo@ugto.mx (M.-L.L.-d.-l.-V.-M.); yeni.rn@hotmail.com (Y.R.-N.); 2 Department of Medicine and Nutrition, Health Sciences Division, University of Guanajuato, Leon Campus, Leon de los Aldama 37320, Mexico; mdc.preciadopuga@ugto.mx (M.-d.-C.P.-P.); rosy_gr@hotmail.com (J.-R.G.-R.); 3 Department of Surgery, General Hospital Leon, Leon de los Aldama 37320, Mexico; benjajordan1@gmail.com (B.J.-P.); seragarnelo@hotmail.com (S.G.-C.)

**Keywords:** NAFLD diagnosis, predictive models, gallstone disease, sex-specific variations

## Abstract

(1) Background: Evidence regarding Non-Alcoholic Fatty Liver Disease (NAFLD) diagnosis is limited in the context of patients with gallstone disease (GD). This study aimed to assess the predictive potential of conventional clinical and biochemical variables as combined models for diagnosing NAFLD in patients with GD. (2) Methods: A cross-sectional study including 239 patients with GD and NAFLD diagnosed by ultrasonography who underwent laparoscopic cholecystectomy and liver biopsy was conducted. Previous clinical indices were also determined. Predictive models for the presence of NAFLD stratified by biological sex were obtained through binary logistic regression and sensitivity analyses were performed. (3) Results: For women, the model included total cholesterol (TC), age and alanine aminotransferase (ALT) and showed an area under receiver operating characteristic curve (AUC) of 0.727 (*p* < 0.001), sensitivity of 0.831 and a specificity of 0.517. For men, the model included TC, body mass index (BMI) and aspartate aminotransferase (AST), had an AUC of 0.898 (*p* < 0.001), sensitivity of 0.917 and specificity of 0.818. In both sexes, the diagnostic performance of the designed equations was superior to the previous indices. (4) Conclusions: These models have the potential to offer valuable guidance to healthcare providers in clinical decision-making, enabling them to achieve optimal outcomes for each patient.

## 1. Introduction

Non-Alcoholic Fatty Liver Disease (NAFLD) is a term that covers all grades and stages of the pathological condition in which ≥ 5% of hepatocytes undergo macrovesicular steatosis, in the absence of any previously identified alternative cause of steatosis. The range of NAFLD spans from simple steatosis, characterized by hepatic fat accumulation that may be accompanied by mild inflammation, to Non-Alcoholic Steatohepatitis (NASH), which is further characterized by inflammation, cellular injury and cirrhosis [1].

It is currently estimated that the overall prevalence of NAFLD is approximately 25% worldwide, with over 80 million people affected only in the United States [2]. Regarding NASH, the global prevalence has been estimated to range from 3% to 5% [3].

Due to its association with metabolic syndrome, NAFLD is present in up to 80% of people with obesity [4]. Mexico is one of the countries with the highest prevalence of obesity, with 36.1% of the adult population presenting this condition, and thus, an elevated prevalence of metabolic syndrome of 41% [5]. The prevalence of NAFLD in Hispanic people of Mexican origin in the USA is higher (33%) than in the rest of the Hispanic population in that country, even after adjusting for the constituent variables of metabolic syndrome [6]. Liver biopsy is the gold standard for NAFLD diagnosis; hence, the overall prevalence could be underestimated due to reports with other diagnostic methods with lower efficacy, such as ultrasonography [7]. Several indices and models based on conventional markers have been proposed for the diagnosis of NAFLD; however, none have surpassed the diagnostic capacity of liver biopsy. Some indices associated with obesity and metabolic dysfunction proposed for the evaluation of patients with probable NAFLD include the triglyceride–glucose index (TyG index), triglyceride glucose–body mass index (TyG-BMI), triglyceride to high-density lipoprotein cholesterol ratio (TG/HDL), alanine aminotransferase aspartate aminotransferase ratio (ALT/AST ratio) and hepatic steatosis index (HSI), among others [8]. 

Gallstone disease (GD) is a term that groups patients with cholecystolithiasis or those who have previously undergone cholecystectomy and is one of the most common disorders of the gastrointestinal tract [9]. The high prevalence of both GD and NAFLD increases the probability of their coexistence. Furthermore, these two conditions share common risk factors, such as overweight/obesity, hypertriglyceridemia, insulin resistance and type 2 diabetes mellitus. Their simultaneous occurrence might also be influenced by shared underlying mechanisms, a phenomenon that has been previously documented [10]. However, information regarding the specific factors involved in NAFLD development in the setting of patients undergoing cholecystectomy with gallstone disease diagnosis in a high-risk population, such as the Mexican population, is scarce. 

The main contribution of this paper is the design of two different models for the prediction of NAFLD, considering biological sex, based on clinical and biochemical variables, with the potential to offer a possible non-invasive diagnostic tool. This study aimed to assess the predictive potential of conventional clinical and biochemical variables as combined models for diagnosing NAFLD, specified by biological sex in patients with GD undergoing cholecystectomy.

## 2. Materials and Methods

A cross-sectional study including 239 patients diagnosed with gallstone disease and NAFLD by ultrasonography who underwent laparoscopic cholecystectomy and liver biopsy was performed in order to obtain a definitive diagnosis of NAFLD. Laparoscopic liver biopsy is a safe procedure with the added advantage of obtaining adequate tissue sampling under direct vision [11]. Participants were recruited in the General Hospital of the City of Leon, Guanajuato, Mexico. Previous signed and informed consent was obtained from each patient. The study was approved by the Ethics Committee of the General Hospital of Leon (HGL-SSGTO00075) and by the Institutional Committee of Bioethics in Research of the University of Guanajuato (CIBIUG-P03-2017), which regulates risk greater than the minimum according to the regulations of the General Health Law on Research for Health of the United Mexican States in force according to article 17. The procedures of this study were carried out following the guidelines established internationally in the Declaration of Helsinki (2013) by the World Medical Association, the General Health Law and the Regulation of the General Health Law on Research for Health of the United Mexican States.

### 2.1. Patient Selection 

Patients with gallstone disease and NAFLD diagnosis by ultrasonography undergoing laparoscopic cholecystectomy were included in the study, from whom a liver tissue sample was obtained to confirm the NAFLD diagnosis. The participants had no significant alcohol consumption (more than 20 g/day in women and more than 30 g/day in men) [12]. Patients with previously known liver disease (alcoholic liver disease, hepatitis of any cause, cirrhosis, carcinoma, sclerosing cholangitis); secondary hepatic steatosis caused by the intake of medications, such as glucocorticoids, methotrexate, amiodarone, tamoxifen and some agent antivirals; or secondary to intense and rapid weight loss, other metabolic alterations or inflammatory bowel diseases were not included in the study.

### 2.2. Clinical Assessment 

Clinical data, body mass index (BMI) and results of biochemical variables, which have previously been linked to NAFLD [13,14,15], were taken directly from each patient’s clinical record. The biochemical variables that were collected were as follows: glucose, alkaline phosphatase (ALP), total bilirubin (TB), direct bilirubin (DB), indirect bilirubin (IB), high-density lipoprotein (HDL), total cholesterol (TC), triglycerides (TG), aspartate aminotransferase (AST) and alanine aminotransferase (ALT), which were determined by standard colorimetric methods with a chemistry analyzer (Auto KEM II, Kontrollab, Italy) in the General Hospital of Leon clinical laboratory. Moreover, clinical indices such as TyG index; TyG-BMI; TG/HDL ratio; ALT/AST ratio; and HSI were determined in every patient.

### 2.3. Ultrasonographic Diagnosis of NAFLD and Cholelithiasis 

An abdominal ultrasound of the liver and biliary tract was performed using Philips HD7 Ultrasound Machine (Washington, DC, USA), with a convex multifrequency transducer from 2 to 5 MHz. Transabdominal ultrasonography remains the first line of radiological investigation to NAFLD and cholelithiasis due to its high sensitivity and specificity (85% and 93%, respectively, for NAFLD and 88 and 80%, respectively, for choleltithiasis) [16,17].

The diagnosis of gallstone disease was established by abdominal ultrasonography (US) in the presence of one of the following criteria: (I) US evidence of gallstones: one or more echogenic, distally shadowing, possibly movable structures in the gallbladder; (II) echogenic material within the gallbladder fossa with constant shadowing with little or no visualization of the gallbladder [9].

### 2.4. Non-Alcoholic Fatty Liver Histopathological Diagnosis

The liver biopsy was taken from segment V of the right hepatic lobe, due to its proximity to the gallbladder and evidence of preferential fat storage in the right hepatic lobe [18,19,20]. 

Histopathological NAFLD diagnosis was made based on the Kleiner scoring system and activity index (NAS) [21] by an experienced pathologist. The NAFLD activity score (NAS) combines histological characteristics to offer a global and structured evaluation of the severity and progression of the disease. The scale evaluates the degree of steatosis (0–3), hepatocyte ballooning (0–2) and lobular inflammation (0–3), whose weighted sum ranges from 0 to 8 [21]. Cases with NAS 0-2 were classified as non-NAFLD, and patients with a score ≥ 3 were diagnosed with the disease. 

### 2.5. Statistical Analysis 

Data normality was assessed using the Kolmogorov–Smirnov statistical test. To compare means between non-NAFLD and NAFLD patients, Student’s *t*-test and Mann–Whitney U-test were employed based on the specific distribution of variables. Furthermore, categorical variables were analyzed using the Chi-squared (χ^2^) test. Subsequently, the clinical and biochemical variables (excluding the indices and previously determined scores) with statistically significant differences between groups were introduced into a binary logistic regression model for the presence or absence of NAFLD in specific groups by biological sex. The used method was Backward Conditional, in which the variables in the last step were selected for the final regression equation, this in the mathematical framework of Bayes’ conditional law. Subsequently, from the previous indices and equations and the new models designed for each sex, the areas under the receiver operating characteristic curves (AUROC) were calculated; diagnostic parameters such as sensitivity, specificity, positive predictive value and negative predictive value were also determined. The optimal cut-off value was selected based on the maximum value of Youden’s Index. Statistical significance was considered using a *p* value < 0.05. All statistical analyses were carried out using SPSS (Statistical Package for the Social Sciences) IBM version 25.0 (Chicago, IL, USA).

## 3. Results

A total of 239 patients with GD who underwent cholecystectomy were included in this study, comprising 37 (15.5%) men and 202 (84.5%) women. Regarding NAFLD diagnosis, 78 (32.6%) patients were diagnosed with the disease, and they had a median age of 44 years and an average BMI of 30.25 ± 5.3. Notably, 65 (83%) of the NAFLD patients were women. Most NAFLD cases had stage 1 in every histological parameter, with 39% with steatosis, 68.8% with inflammation, 92.2% with ballooning and 54.5% with fibrosis. There were no cases with severe fibrosis (score > 2). Table 1 shows the differences in clinical and biochemical parameters between the non-NAFLD and NAFLD groups. Patients with NAFLD diagnosis showed a higher age, higher serum triglyceride levels and higher BMI compared to subjects without the disease. Serum glucose, AST and ALT levels were higher in patients with NAFLD as well. Obesity and lipids-related indices were calculated, and the TyG index, TyG-BMI and TG/HDL ratio were significantly superior in NAFLD patients. On the other hand, the Hepatic Steatosis Index and the ALT/AST ratio were not statistically different in both groups.

Next, based on the statistically significant parameters identified in the first analysis, logistic regression models were conducted to assess the presence or absence of NAFLD; these are shown in Table 2. In the female population, the model included serum cholesterol, age and ALT. The equation obtained was y = 0.038 × Age + 0.046 × ALT + 0.557 × TC − 5.722. In contrast, for men, the included variables were TC, BMI and AST levels and the corresponding equation was y = 1.681 × TC + 0.545 × BMI + 0.029 × AST − 24.893. 

To assess the association with NAFLD of the previous indices and equations, as well as the designed models stratified by biological sex (Table 3), a univariable logistic regression analysis was conducted where the previous indices showed differential behaviors in men and women. In the female group, the TyG index, TyG-BMI and TG/HDL ratio showed significant associations with the disease, as well as the designed model. On the other hand, this behavior varied in the male group, where only the TyG-BMI index and the designed model for men maintained a significant association. 

**Figure 1:**
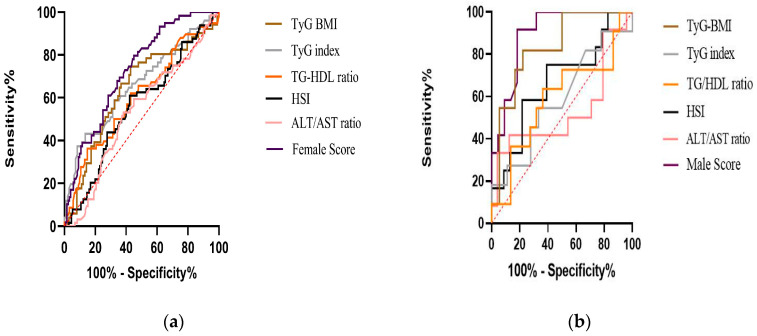
ROC curve analysis illustrating the diagnostic performance of the previous indices and scores, and the new models stratified by biological sex. (**a**) Female subjects. (**b**) Male subjects.

In relation to the diagnostic performance of the previous indices and equations, as well as the model specifically designed for the female population, the TyG Index, TyG-BMI and TG/HDL ratio showed statistically significant AUCs. Moreover, the model for the female group exhibited the highest AUC with a value of 0.727 (*p* < 0.001); this model showed a sensitivity of 0.831 and a specificity of 0.517 (Table 4) (Figure 1a).

On the other hand, in men, the TyG-BMI index and the Male NAFLD Score showed significant diagnostic performance. The scale designed for men had an AUC of 0.898 with high values of sensitivity and specificity at 0.917 and 0.818, respectively (Table 5) (Figure 1b).

## 4. Discussion

The high prevalence of NAFLD among the Hispanic population, along with its significant impact on healthcare systems, emphasizes the urgent need for the identification of diagnostic models capable of predicting the presence of the disease, which would thus facilitate early diagnosis and effective prognosis stratification for each patient. Furthermore, the presence of comorbidities, such as GD, could cause variations in the patterns of the variables associated with NAFLD. Based on the fact that NAFLD is a sexually dimorphic disease, the factors involved in its pathogenesis and progression may have different roles and grades of impact in men and women [22]. 

Previous evidence has shown that women bear a lower risk of developing NAFLD [23]. Nevertheless, in the context of GD, a previous study reported an increased risk of NAFLD in women [24]. In accordance with this observation, in our cohort, there was a higher proportion of female patients with NAFLD (83.9%), which is similar to previous studies among patients undergoing cholecystectomy [25,26]. This has been explained by the role of estrogens in stimulating the liver’s release of biliary cholesterol, which leads to an increase in cholesterol saturation in bile [27]. 

Several models based on conventional biomarkers have been proposed for the evaluation of patients with probable NAFLD; however, liver biopsy has not yet been replaced for the definitive diagnosis of this condition. In our cohort, the TyG index, TyG-BMI, TG/HDL ratio, ALT/AST ratio and HSI were determined for each patient and differential results were obtained for men and women. In the female population, only the TyG index, TyG-BMI and TG/HDL ratio showed a significant association with the disease; however, their diagnostic accuracy was low. In contrast to these results, the high diagnostic accuracy of the TyG index and TyG-BMI has been previously reported in a Chinese population, where patients exhibited with AUCs above 0.90 [8]. 

On the other hand, in male patients, among the previous indices, only the TyG-BMI maintained a significant association and a high AUC value of 0.833, which is consistent with previous reports [8]. 

The HSI was designed in a Korean population [28], and some studies report excellent prediction of NAFLD [29]. However, in our cohort, it did not show a significant association with the presence of the disease, which is consistent with previous reports in Mexican patients [30].

In our study, two different models for NAFLD prediction, accounting for biological sex, were designed based on clinical and biochemical variables. In female patients, the model showed the most significant ability to discriminate NAFLD cases compared to the previous scores applied, using age, TC and ALT levels. These factors have been extensively associated with NAFLD presentation and progression. However, many studies have assessed them individually for NAFLD prediction, instead of combining them in a predictive model, in a sex-differential manner. In the general population, the prevalence of NAFLD increases with age and so does the likelihood of NASH progression [2,31]. Nevertheless, in the context of patients with GD there are discrepancies. While some authors report that subjects with the disease tend to be older than controls [26], other studies have not identified associations between NAFLD and age in patients with GD [9].

Another variable included in the model was TC. It has been reported that alterations in cholesterol homeostasis are also present in NAFLD patients, and these abnormalities lead to oxidative stress, mitochondrial dysfunction and endoplasmic reticulum (ER) stress. This triggers the activation of hepatic Kupffer cells and hepatic stellate cells (HSCs), resulting in increased inflammation and fibrosis [32]. Regarding ALT, included in the female model, it is documented that it rises in patients with NAFLD, reflecting liver damage. Furthermore, elevated ALT correlates with NAFLD progression, and its serum levels are used in the clinical context as a marker of liver disease [33].

In the male population, the model included TC, BMI and AST levels. The model showed the strongest prediction capacity, with an AUC of 0.898, a sensitivity of 0.917 and a specificity of 0.818. TC was the only common variable for men and women, reiterating its implications in NAFLD. Obesity, characterized by an increased production of cytokines and insulin resistance (components of metabolic syndrome), disrupts lipid homeostasis and induces oxidative stress. This alteration in the metabolic balance increases the risk of developing NASH [32]. Obesity is a known risk factor for the development of NAFLD [25]. A meta-analysis reported a pooled overall obesity prevalence among NAFLD patients and NASH patients of 51.34% and 81.83%, respectively, which supports the role of obesity in the progression of the disease to NASH and further stages [2,34].

Despite the previously described role of obesity in NAFLD, there are contradictory reports regarding the association between NAFLD and BMI according to biological sex. It has been reported that BMI was independently associated with NAFLD in both men and women in the Chinese population [33]. However, in a study of the Indian population, BMI was found to be strongly associated with fatty liver only in men [35], which is similar to what we observed in the Mexican population. This supports the role of BMI in the assessment of NAFLD in men with GD. As for women, other methods useful to determine visceral fat could be taken into consideration in order to achieve a better approach. Another variable included in the male predictive model was AST. Previous reports have shown differences in transaminases levels according to its presence [26] and the disease severity [30]. However, serum transaminase levels have shown highly variable sex-specific behavior in previous reports. A study carried out in mouse genetic models of NAFLD reported that serum AST and ALT levels were significantly higher in 3-month-old male mice compared to female ones [36]. Several studies in humans have also revealed transaminase differences between men and women; however, the results have been conflicting [23,33]. In our cohort, these differences could be influenced by the presence of GD as an underlying condition, which may produce additional liver abnormalities, resulting in differential patterns of transaminase levels.

To the best of our knowledge, this is the first study that has developed straightforward and practical predictive models using conventional variables for diagnosing NAFLD in Mexican patients with gallstone disease, stratified by biological sex and with NAFLD confirmatory diagnosis by liver biopsy. These models have the potential to offer valuable guidance to healthcare providers in clinical decision-making, enabling them to achieve optimal outcomes for each patient. The clinical value of this and related research could significantly contribute to the development of predictive models for NAFLD using artificial neural networks in the medium term. These models could be integrated into new, easily accessible devices, thereby supporting the diagnosis and timely management of patients.

One limitation of our study is that we did not consider other variables that could be relevant, such as waist circumference, levels of low- and very low-density lipoproteins and the assessment of insulin function and sensitivity via methods like the Homeostatic Model Assessment of Insulin Resistance (HOMA-IR). The incorporation of these variables could potentially enhance the diagnostic capability of these predictive models, given their known associations with metabolic syndrome and Non-Alcoholic Fatty Liver Disease (NAFLD). This paves the way for future investigations aimed at refining and optimizing the predictive efficacy of these models.

The evaluation of patients with NAFLD should also consider biological sex. Although clinical and biochemical variables have been extensively associated with the disease, our study reveals significant variations between men and women that may have pathophysiological implications. Future research aimed at developing predictive models for NAFLD should incorporate sex-specific analysis to achieve more clinically applicable results.

## Figures and Tables

**Table 1:** Baseline characteristics of patients undergoing cholecystectomy according to NAFLD diagnosis.

Variables	Non-NAFLD (*n* = 161)	NAFLD (*n* = 78)	*p*-Value
Age (years)	35 (16)	44(15)	<0.001
Biological sex			0.724
Female (%)	137 (85)	65 (83)	
Male (%)	24 (15)	13 (17)	
BMI (kg/m^2^)	28.32 ± 5.3	30.25 ± 5.3	0.009
Glucose (mmol/L)	5.1 (0.84)	5.22 (1.38)	0.016
TC (mmol/L)	4.1 ± 0.8	4.5 ± 0.9	0.001
HDL (mmol/L)	1.09 ± 0.28	1.18 ± 0.31	0.259
TG (mmol/L)	1.38 (0.87)	1.74 (1.6)	0.002
TB (µmol/L)	9.06 (5.13)	9.7 (5.13)	0.818
DB (µmol/L)	3.42 (3)	5.13 (4.7)	0.285
IB (µmol/L)	5.13 (4.6)	5.13 (4.02)	0.282
AST (IU/L)	21 (10.9)	28 (15.68)	<0.001
ALT (IU/L)	24 (15.38)	29 (23)	0.001
ALP (IU/L)	88 (32.78)	87.5 (35.82)	0.918
TyG	8.58 ± 0.52	8.91 ± 0.6	<0.001
TyG-BMI	244.7 ± 49.6	274.4 ± 57.9	<0.001
TG/HDL ratio	1.29 (1.15)	1.62 (1.74)	0.024
ALT/AST ratio	1.2 ± 0.6	1.14 ± 0.4	0.447
HSI	39.6 ± 6.8	41.2 ± 6.18	0.109

Note: BMI, body mass index; TC, total cholesterol; HDL, high-density lipoproteins; TG, triglycerides; TB, total bilirubin; DB, direct bilirubin, IB, indirect bilirubin; AST, aspartate aminotransferase; ALT, alanine aminotransferase; ALP, alkaline phosphatase; TyG index, triglyceride–glucose index; TyG-BMI, triglyceride glucose–body mass index; TG/HDL, triglyceride to high-density lipoprotein cholesterol ratio; ALT/AST ratio, alanine aminotransferase aspartate aminotransferase ratio; HSI, hepatic steatosis index. Data are presented as number (%), mean ± SD or median (IQR). Student’s *t*-test or Mann–Whitney U-test to obtain *p*-value according to particular distribution of variables; Chi-squared (χ^2^) test.

**Table 2:** NAFLD prediction scores design stratified by biological sex.

Models		B	Standard Error	*p*-Value	OR	Confidence Interval	
						Lower	Upper
Female NAFLD Score	TC	0.557	0.251	0.026	1.745	1.068	2.852
	Age	0.038	0.017	0.029	1.038	1.004	1.074
	ALT	0.046	0.015	0.002	1.051	1.017	1.079
	Constant	−5.722	1.222	<0.001	0.004		
Male NAFLD Score	TC	1.681	0.983	0.087	5.371	0.782	36.865
	BMI	0.545	0.266	0.040	1.725	1.024	2.905
	AST	0.029	0.021	0.171	1.030	0.987	1.074
	Constant	−24.893	10.935	0.023	0		

Note: BMI, body mass index; TC, total cholesterol; AST, aspartate aminotransferase; ALT, alanine aminotransferase.

**Table 3:** Univariable logistic regression analysis for prediction of NAFLD stratified by biological sex.

		Female		Male	
		OR (95% CI)	*p*-Value	OR (95% CI)	*p*-Value
Previous equations	TyG index	3.067 (1.582–5.946)	0.001	2.014 (0.534–7.603)	0.302
	TyG-BMI	1.009 (1.002–1.015)	0.012	1.038 (1.005–1.072)	0.023
	TG/HDL ratio	1.375 (1.038–1.822)	0.026	1.264 (0.687–2.324)	0.451
	ALT/AST ratio	0.855 (0.487–1.503)	0.586	0.553 (0.118–2.580)	0.451
	HSI	1.021 (0.975–1.069)	0.375	1.120 (0.992–1.263)	0.066
New equations	Female NAFLD Score	1.891 (1.371–2.609)	<0.001	NA	
	Male NAFLD Score	NA		2.365(1.301–4.3)	0.005

Note: TyG index, triglyceride–glucose index; TyG-BMI, triglyceride glucose–body mass index; TG/HDL, triglyceride to high-density lipoprotein cholesterol ratio; ALT/AST ratio, alanine aminotransferase aspartate aminotransferase ratio; HSI, hepatic steatosis index; NA, non-applicable.

**Table 4:** Performance of indices and scores in NAFLD prediction in female subjects.

		AUC (95% CI)	Threshold	Sensitivity	Specificity	PPV	NPV	*p*-Value
Previous equations	TyG index	0.658 (0.562–0.754)	9.0064	0.431	0.865	0.4	0.741	0.002
	TyG-BMI	0.651 (0.554–0.748)	253.25	0.745	0.583	0.307	0.811	0.003
	TG-HDL ratio	0.602 (0.511–0.693)	2.1202	0.373	0.844	0.452	0.735	0.027
	ALT/AST ratio	0.531 (0.445–0.617)	1.1673	0.531	0.614	0.416	0.729	0.480
	HSI	0.557 (0.472–0.643)	40.572	0.609	0.573	0.392	0.75	0.194
New equation	Female NAFLD Score	0.727 (0.652–0.802)	−0.9901	0.831	0.517	0.423	0.861	<0.001

Note: AUC, area under receiver operating characteristic curve; PPV, positive predictive value; NPV, negative predictive value; CI, confidence interval; TyG index, triglyceride–glucose index; TyG-BMI, triglyceride glucose–body mass index; TG/HDL, triglyceride to high-density lipoprotein cholesterol ratio; ALT/AST ratio, alanine aminotransferase aspartate aminotransferase ratio; HSI, hepatic steatosis index.

**Table 5:** Diagnostic performance of indices and scores in NAFLD prediction in male subjects.

		AUC (95% CI)	Threshold	Sensitivity	Specificity	PPV	NPV	*p*-Value
Previous equations	TyG index	0.576 (0.351–0.8)	8.8395	0.545	0.66	0.4	0.705	0.5
	TyG-BMI	0.833 (0.683–0.984)	262.888	0.818	0.78	0.523	0.875	0.003
	TG/HDL ratio	0.591 (0.372–0.810)	2.026	0.667	0.636	0.473	0.778	0.401
	ALT/AST ratio	0.469 (0.241–0.697)	1.4477	0.5	0.708	0.545	0.73	0.763
	HSI	0.667 (0.471–0.862)	41.172	0.583	0.783	0.571	0.78	0.11
New equation	Male NAFLD Score	0.898 (0.796–1)	−1.075	0.917	0.818	0.67	0.94	<0.001

Note: AUC, area under receiver operating characteristic curve; PPV, positive predictive value; NPV, negative predictive value; CI, confidence interval; TyG index, triglyceride–glucose index; TyG-BMI, triglyceride glucose–body mass index; TG/HDL, triglyceride to high-density lipoprotein cholesterol ratio; ALT/AST ratio, alanine aminotransferase aspartate aminotransferase ratio; HSI, hepatic steatosis index.

## Data Availability

The data associated with the paper are available from the corresponding author on reasonable request.
